# 

**Published:** 1891-05

**Authors:** 


### LITERARY.

### TOPOLOBAMPO AND CO-OPERATION.

HALL'S JOURNAL OF HEALTH is so progresssive, so true to humanity, and, in fact, so true to truth itself, that I am always ready to commend it instead of uttering any objection to its subject matter, but from its brief reference to the Credit Foncier Company in a disparaging way, I was led to think that it is not acquainted with the latest facts of the case.

The Credit Foncier Company, which carried out its high aims for humanity by settling in Sinaloa, Mexico, on the Pacific coast, in 1886, made some serious mistakes and passed through a good deal of hardship for a time, but the tide has turned in their favor, and they seem to be going on to power and fortune. I

have seen several letters from there which speak in very warm terms of the climate, soil, the healthfulness, the character of the colonists and the prospects generally. A late accession from Kansas, headed by the banker Hoffman, has increased their numbers to 330, and other companies are coming. A twenty mile ditch is being constructed from the Fuerte river to Topolobamo, which, among other things, will irrigate 60,000 acres of the lands of the colonists. The Mexican Government has made fine concessions to them, and the Credit Foncier Company has taken the contract to construct and lease the Mexican Western Railroad from Topolobampo harbor to Galveston, Texas, 1,100 miles. A number of weeks since the newspapers gave an account of the paying of $30,000 to the Mexican government, by Mr. Albert K. Owen, the founder of the colony. On all sides, the colonists are moving on, not rapidly, but surely, and they are more happy than ever in the belief that they are to practicalize a system of fraternity and co-operation that shall yet bless the world.

E. D. BABBITT, M. D.

New York College of Magnetics, 78 East Tenth street.

---

THE NEW SOUTH.—One of the most enterprising towns which has awakened to renewed life and almost unexampled energy, since the new order of things in the old Dominion State, is Staunton, nestled in one of the most picturesquely beautiful sections of the famous Shenandoah Valley, which burst upon our northmen in the late conflict of arms, like the realization of a long cherished dream, and not a few of them have realized the still happier dream, by making it their home now that the sword has given place to the plow share and the pruning hook is busy on the vine clad hills.

Staunton is a legitimate outgrowth of the business like sagacity and enterprise of men and women, who recognize that intelligent labor of hand and brain is the legitimate source of human advancement. The beautifully illustrated Album of the Staunton Development Co., whose Eastern office, managed by D. Z. Evans, Jr., Esq., is in Philadelphia, would do ample credit to any of the older and wealthier of our commercial centers. Surrounded by some of the richest mineral deposits to be found in our available mining regions, Staunton has been free to encourage all manner of industry, and is now harvesting a well earned reward. Her public and private edifices are a credit to the artistic taste and skill of her people.

THE DISEASES OF PERSONALITY. By Prof. T. H. Ribot, Editor *Revue Philosophique*, Paris, France.

This is a work of very great value to the student of human nature, compassing all real and imaginary disorders resulting from an unbalanced condition of the members and faculties which in their healthy unification constitute the selfhood of man. The scope of the work embraces every known mental disease from harmless hallucination to a state of downright madness, with an explanation of the causes, hereditary and otherwise, which lie at their foundation. The author recognizes the dual character of man—the physical and psychological—which conjointly make up the ego, or personality of our being, and the necessity of their harmonious relations—master and machine—in order to a state of perfect health. The chapters which treat of Emotional Disorders are particularly interesting and instructive.

The Open Court Publishing Co., Chicago. Price, 75c.

HELPS FOR HOME NURSING. By H. Ovington ; Chas. H. Kerr & Co., Chicago, publishers.

This handy little volume of 114 pp., is destined to be a valuable assistant to those of the household, whose varied duties include sometimes the care of the sick. Nothing seems to have been lost sight of to make it a complete manual for the sick room, and with this little book in hand, the most inexperienced attendant would find the way clear for the discharge of every duty. For sale by the publishers, price 50 cts.

We are in receipt of the THIRTEENTH ANNUAL REPORT of the Presbyterian Eye, Ear and Throat Charity Hospital, Baltimore. A most excellent showing for the year 1890. All such institutions are a blessing to the race and deserve a liberal patronage.

HOW TO MAGNETIZE.—By James Victor Wilson. Fowler & Wells Co., New York, publishers.

This handy pamphlet of 104 pp., conveys much valuable information upon a subject too little understood and too liable to abuse to be experimented with by novices, and clearly defines the relation between mesmerism and subject, introducing many facts of historical interest. Price 25 cts.

